# A comprehensive analysis of Wnt/β-catenin signaling pathway-related genes and crosstalk pathways in the treatment of As_2_O_3_ in renal cancer

**DOI:** 10.1080/0886022X.2018.1456461

**Published:** 2018-04-10

**Authors:** Yan-Lei Li, Yu-Fen Jin, Xiu-Xia Liu, Hong-Jun Li

**Affiliations:** a Medical Examination Center, China-Japan Union Hospital of Jilin University, Changchun, China;; b Clinical Laboratory, The Second Hospital of Jilin University, Changchun, China

**Keywords:** As_2_O_3_, renal cancer, differentially expressed gene, Wnt signaling pathway, therapeutic targets

## Abstract

We aimed to investigate the effect of As_2_O_3_ treatment on Wnt/β-catenin signaling pathway-related genes and pathways in renal cancer. Illumina-based RNA-seq of 786-O cells with or without As_2_O_3_ treatment was performed, and differentially expressed genes (DEGs) were identified using Cuffdiff software. TargetMine was utilized to perform Gene Ontology (GO) pathway and Disease Ontology enrichment analyses. Furthermore, TRANSFAC database and LPIA method were applied to select differentially expressed transcription factors (TFs) and pathways related to Wnt/β-catenin signaling pathway, respectively. Additionally, transcriptional regulatory and pathway crosstalk networks were constructed. In total, 1684 DEGs and 69 TFs were screened out. The 821 up-regulated DEGs were mainly enriched in 67 pathways, 70 GO terms, and 46 disease pathways, while only 1 pathway and 5 GO terms were enriched for 863 down-regulated DEGs. A total of 18 DEGs (4 up-regulated and 14 down-regulated genes) were involved in the Wnt/β-catenin signaling pathway. Among the 18 DEGs, 4 ones were TFs. Furthermore, 211 pathways were predicted to be linked to the Wnt/β-catenin signaling pathway. In conclusion, As_2_O_3_ may have a significant effect on the Wnt/β-catenin signaling pathway for renal cancer treatment. The potential key DEGs are expected to be used as therapeutic targets.

## Introduction

Renal cell carcinoma (RCC) is one of leading malignant condition in both males and females, with 62,700 estimated new cases and 14,240 estimated deaths in the United States in 2016 [1]. The treatment of RCC has been greatly improved over the past 15 years due to the advanced genomics and biological discoveries [2]. However, more effective forms of therapies are needed that will bring benefits for a higher percentage of patients.

Wnt family consists of 19 secreted ligands enriched by cysteine, which is essential for multiple developmental and physiological events, including proliferation, differentiation, migration, death, and polarity [3], and affects multiple intracellular signaling cascades, including the β-catenin-dependent and -independent pathways [4]. Furthermore, increasing evidences reveal that Wnt components are shared by other signaling pathways and crosstalk occurs between Wnt signaling with many other pathways, such as transforming growth factor β (TGF-β)-signaling pathway and fibroblast growth factor (FGF) pathways [5]. Notably, activated Wnt signaling pathway is frequently observed in early stages of many tumors, most tissues that normally depend on Wnt for repair or self-renewal [6]. Meanwhile, previous studies have demonstrated that multiple genes or proteins can affect the occurrence and development of renal cancer by targeting Wnt signaling pathway. For instance, Gnemmi et al. [7] have found that MUC1 drives epithelial–mesenchymal transition (EMT) in renal cancer through Wnt/β-catenin pathway. *UBE3C* promotes growth and metastasis of RCC via activating Wnt/β-Catenin pathway [8]. Besides, knockdown of *MALAT1* reduces expression of proteins in Wnt/β-Catenin pathway in RCC cell lines [9]. RCC survival is suppressed in part due to inhibition of Wnt/β-catenin signaling by ethacrynic acid, ciclopirox olamine, and piroctone olamine [10]. These studies suggest that the development of therapies targeting the Wnt signaling pathway may provide novel and effective treatment options for renal cancer patients.

Arsenic trioxide (As_2_O_3_), as a traditional Chinese medicine, has been identified to play an important role in the research and treatment for cancers, such as acute breast cancer, acute promyelocytic leukemia, gastric cancer, neuroblastoma, and esophageal carcinoma [11]. It has reported that As_2_O_3_ induces apoptosis by reducing the activities of nuclear factor-κB (NF-κB) and human telomerase reverse transcriptase (hTERT) [12]. Recently, the role of As_2_O_3_ in renal cancer has also been reported, and As_2_O_3_ with amino acids can be used to treat renal cancer by down-regulating Bcl-2 and induce the apoptosis of renal cancer 786-O cells through up-regulating the expression of Bax [13]. However, the molecular mechanism of As_2_O_3_ treatment on renal cancer is still far from completely clear. Studies have shown that the Wnt signaling pathway plays critical roles in cell apoptosis, and some drugs or molecules affect apoptosis process in multiple cancers by targeting Wnt signaling pathway [14]. Therefore, we suspect that Wnt signaling pathway may play pivotal roles in the As_2_O_3_ treatment on renal cancer.

In order to certify our suspicion, the differentially expressed genes (DEGs) were screened after As_2_O_3_ treatment in 786-O renal carcinoma cell lines using bioinformatics methods. The Wnt signaling pathway-related genes, transcription factors (TFs), and crosstalk pathways were also explored in order to extend our understanding of the molecular mechanisms of As_2_O_3_ treatment on renal cancer and provide new insights for the further treatment of renal cancer.

## Methods

### Cell line and cell culture

Renal carcinoma cell line 786-O was purchased from the Cell Bank of Shanghai Institutes for Biological Sciences (Shanghai, China). The cell line was cultivated in Dulbecco’s modified Eagle’s medium (DMEM, GIBCO BRL, Gaithersburg, MD) supplemented with 10% fetal bovine serum (FBS, GIBCO BRL, Gaithersburg, MD), 100 IU/mL penicillin and 100 μg/mL streptomycin and cultured in a humidified 37 °C incubator containing 5% CO_2_.

### Grouping and cell treatment

Two groups were set up for this study, namely control groups and As_2_O_3_ groups, with two samples for each group. According to the result of tetrazolium (MTT) assay, in which the 786-O cells were treated by different concentration of As_2_O_3_ (0, 0.5, 1, 2, 3, 5, 6 and 8 µmol/L), 2 µmol/L As_2_O_3_ was used to treat 786-O cells in As_2_O_3_ group due to higher cell viability. Thus, the cells in As_2_O_3_ group were exposure to 2 µmol/L As_2_O_3_ (Sigma, St Louis, MO) for 48 h. Equal volume of DMEM (GIBCO BRL, Gaithersburg, MD) was added to the control groups.

### RNA isolation and sequencing

Total RNA of the four cell line samples were extracted using Trizol reagent (GIBCO BRL, Gaithersburg, MD) according to the manufacturer’s instructions, respectively. RNA sequencing was then performed using an Illumina HiSeq 2000 (Nanjing Vazyme Biotech Co., Ltd., Nanjing, China).

### Quality control and mapping RNA-seq reads to reference genome

Fastx_toolkit (Version 0.0.13 from Assaf Gordon Hannon Lab) was utilized for quality control, and scoring systems of Sanger (Phred +33) was chosen for the read values. High-quality data were obtained from raw data by removing adapter and read values <10. Only the reads with length more than 50 after trimmed and the reads with more than 80% of high-quality (Q value ≥20) were selected. The TopHat2 software (http://ccb.jhu.edu/software/tophat/index.shtml) [15] was utilized to map the clean reads to the reference human genome (hg19) with mismatches ≤2. Genomics and refseq annotation files were obtained from the UCSC database (University of California Santa Cruz, http://genome.ucsc.edu/).

### Identification of DEGs

The differential expression analysis of genes between As_2_O_3_ and control groups was performed by using Cuffdiff software (http://cufflinks.cbcb.umd.edu/manual.htm#cuffdiff) [16] with the cutoff criteria of *p* values < .05 and fold change >1.

### Functional and pathway enrichment analyses

In order to obtain further insight into the involvement of DEGs in functional and metabolic pathways, the gene functional classification tool DAVID (Database for Annotation, Visualization and Integrated Discovery) (http://david.abcc.ncifcrf.gov/) [17] was used to perform Gene Ontology (GO) [including biological process (BP), cell component (CC), and molecular function (MF)] and KEGG pathway enrichment analyses. The *p* values were corrected for false discovery rate (FDR) by Holm–Bonferroni [18,19], and FDR <.05 and count ≥2 were set as the cutoff criteria.

### Construction of transcriptional regulatory networks

The differentially expressed TFs were identified from DEGs based on the TRANSFAC database (http://transfac.gbf.de/TRANSFAC/) [20]. Then target genes of Wnt/β-catenin signaling pathway-related TFs were predicted by UCSC database. Transcription regulatory networks were constructed based on the regulatory pairs and visualized by Cytoscape software (http://www.cytoscape.org/) [21].

### Pathway-crosstalk analysis

Latent pathway identification analysis (LPIA) method proposed by Pham et al. [22] was used to analyze the crosstalk pathways related with Wnt/β-catenin signaling pathway. Afterwards, based on DEG annotation, the network composed of crosstalk pathways and related DEGs were visualized by Cytoscape software.

## Results

### Identification of DEGs and enrichment analyses

A total of 1684 DEGs between As_2_O_3_ treatment samples and controls were screened out, including 821 up-regulated genes and 863 down-regulated genes.

According to the GO functional enrichment analysis, the up-regulated genes (e.g., *SMAD3* and *FOSL1*) were significantly enriched in a set of GO terms, such as programmed cell death and protein binding (Table 1). The down-regulated genes were markedly enriched in single-organism cellular process (e.g., *TCF7L1* and *FRAT1*) and protein binding (e.g., *NFATC3* and *TCF7L1*) (Table 2).

**Table 1.** The top 5 Gene Ontology terms with the lowest *p* values in BP, CC, and MF for the up-regulated differentially expressed genes.

Category	ID	Term	*p* value	Count	Genes
GO-BP	GO:0008150	Biological process	1.97E-37	651	*SMAD3*, *FOSL1*, *CCND3*, *DMA*, *LAMA1*…
GO-BP	GO:0009987	Cellular process	1.76E-20	595	*SMAD3*, *FOSL1*, *CCND3*, *GNPDA1*, *PARP2*…
GO-BP	GO:0008152	Metabolic process	2.11E-20	510	*SMAD3*, *FOSL1*, *CCND3*, *MAFF*, *BRD1*…
GO-BP	GO:0012501	Programmed cell death	2.36E-18	135	*SMAD3*, *FOSL1*, *CCND3*, *PARP2*, *ADAM8*…
GO-BP	GO:0044237	Cellular metabolic process	3.96E-18	446	*SMAD3*, *FOSL1*, *MTOR*, *MCM9*, *ARIH1*…
GO-CC	GO:0005622	Intracellular	1.09E-31	598	*SMAD3*, *FOSL1*, *CCND3*, *CCDC137*, *IFIT3*…
GO-CC	GO:0044424	Intracellular part	1.00E-30	593	*SMAD3*, *FOSL1*, *CCND3*, *MAMLD1*, *ABCF2*…
GO-CC	GO:0031974	Membrane-enclosed lumen	1.48E-22	255	*SMAD3*, *FOSL1*, *CENPE*, *POLR3C*, *RGS14*…
GO-CC	GO:0043226	Organelle	1.87E-22	558	*SMAD3*, *FOSL1*, *CCND3*, *GNPDA1*, *PARP2*…
GO-CC	GO:0043233	Organelle lumen	2.35E-22	252	*SMAD3*, *FOSL1*, *POLR3C*, *RGS14*, *NUDC*…
GO-MF	GO:0003674	Molecular function	7.56E-36	660	*SMAD3*, *FOSL1*, *CCND3*, *NFKB2*, *ATP1B3*…
GO-MF	GO:0005488	Binding	3.16E-26	596	*SMAD3*, *FOSL1*, *CCND3*, *ATP1B3*, *NOTCH1*…
GO-MF	GO:0005515	Protein binding	5.83E-18	470	*SMAD3*, *FOSL1*, *CCND3*, *ZNF593*, *NOSIP*…
GO-MF	GO:1901363	Heterocyclic compound binding	4.16E-14	295	*SMAD3*, *FOSL1*, *UBE2T*, *SLC25A5*, *GNL2*…
GO-MF	GO:0097159	Organic cyclic compound binding	6.91E-14	297	*SMAD3*, *FOSL1*, *CENPE*, *POLR3C*, *CAMKK2*…

GO: Gene Ontology; BP: biological process; CC: cell component; MF: molecular function.

**Table 2.** The top 5 Gene Ontology terms with the lowest *p* values in BP, CC, and MF for the down-regulated differentially expressed genes.

Category	ID	Term	*p* value	Count	Genes
GO-BP	GO:0008150	Biological process	1.22E-38	672	*TCF7L1*, *AKT3*, *HDAC6*, *FOXO6*, *BCL2L11*…
GO-BP	GO:0009987	Cellular process	1.62E-22	617	*TCF7L1*, *GSTT1*, *CARD10*, *GUCY1A3*, *ST8SIA5*…
GO-BP	GO:0044763	Single-organism cellular process	1.40E-14	518	*TCF7L1*, *FRAT1*, *PARP3*, *CDH6*, *HMGXB4*…
GO-BP	GO:0044699	Single-organism process	7.33E-14	555	*TCF7L1*, *FRAT1*, *PARP3*, *CDH6*, *HMGXB4*…
GO-BP	GO:0008152	Metabolic process	1.14E-10	492	*TCF7L1*, *CTDSPL*, *GPHN*, *CDKN1C*, *HMG20A*…
GO-CC	GO:0005575	Cellular component	3.95E-19	706	*TCF7L1*, *NFATC3*, *FOXO6*, *BCL2L11*, *FRAT1*…
GO-CC	GO:0031988	Membrane-bounded vesicle	1.77E-08	191	*CDH6*, *OPTN*, *CDH16*, *PDZK1IP1*, *TNK2*…
GO-CC	GO:0031982	Vesicle	6.57E-08	193	*TNK2*, *INADL*, *CTDSPL*, *ATP8A1*, *IFITM3*…
GO-CC	GO:0044421	Extracellular region part	7.05E-08	198	*CDH16*, *PDZK1IP1*, *INADL*, *CTDSPL*, *ATP8A1*…
GO-CC	GO:0098590	Plasma membrane region	1.09E-07	45	*HDAC6*, *CDH16*, *INADL*, *EVC2*, *SLC34A3*…
GO-MF	GO:0003674	Molecular function	1.49E-36	673	*NFATC3*, *TCF7L1*, *AKT3*, *HDAC6*, *FAM27E3*…
GO-MF	GO:0005488	Binding	8.29E-16	583	*NFATC3*, *TCF7L1*, *FAM27E3*, *FOXO6*, *BCL2L11*…
GO-MF	GO:0005515	Protein binding	1.49E-11	455	*NFATC3*, *TCF7L1*, *CDKN1C*, *HMG20A*, *CITED2*…
GO-MF	GO:0043167	Ion binding	1.82E-05	267	*HDAC6*, *CDH6*, *GPC6*, *SGK2*, *ARL4C*…
GO-MF	GO:0046872	Metal ion binding	2.25E-05	191	*HDAC6*, *CDH6*, *CDH16*, *TNK2*, *CTDSPL*…

GO: Gene Ontology; BP: biological process; CC: cell component; MF: molecular function.

Furthermore, the pathway enrichment analysis showed that the up-regulated genes were distinctly enriched in multiple pathways, such as proteasome pathway (e.g., *PSMA7*, *PSMB1*, and *PSMB3*), MAPK signaling pathway (e.g., *FOS* and *GADD45A*), and p53 signaling pathway (e.g., *CASP8* and *CCND3*). Meanwhile, the down-regulated genes were significantly enriched in Wnt signaling pathway (e.g., *NFATC3* and *TCF7L1*), and the pathway of complement and coagulation cascades (e.g., *CD55* and *CFD*) (Table 3).

**Table 3.** The top 5 pathways with the lowest *p* values for the differentially expressed genes.

Category	ID	Term	*p* value	Count	Genes
Up-regulated genes	hsa03050	Proteasome	1.38E-10	11	*PSMA7*, *PSMB1*, *PSMB3*, *PSMB4*, *PSMB6*…
	hsa04010	MAPK signaling pathway	1.98E-07	19	*FOS*, *GADD45A*, *HRAS*, *MAP2K3*, *NFKB2*…
	hsa01100	Metabolic pathways	3.80E-07	44	*ATIC*, *ATP6V0B*, *CDS1*, *NDUFAB1*, *POLD1*…
	hsa05145	Toxoplasmosis	2.73E-06	12	*CASP8*, *ITGA6*, *JAK2*, *LAMB3*, *MAP2K3*…
	hsa04115	p53 signaling pathway	1.93E-05	8	*CASP8*, *CCND3*, *CCNE1*, *G0S2*, *GADD45A*…
Down-regulated genes	hsa04310	Wnt signaling pathway	2.90E-52	14	*FRAT1*, *NFATC3*, *AXIN2*, *TCF7L1*, *CUL1*…
	hsa01100	Metabolic pathways	6.91E-10	52	*ALDH1A1*, *AXIN2*, *DCLK1*, *HOMER2*, *IMPA2*…
	hsa04610	Complement and coagulation cascades	3.70E-08	11	*CD55*, *CFD*, *CFI*, *KLKB1*, *SERPINA1*…
	hsa04146	Peroxisome	8.20E-05	8	*AXIN2*, *MTMR11*, *SOD3*, *SUCLG2*, *TSPAN14*…
	hsa05150	*Staphylococcus aureus* infection	4.25E-04	6	*C3*, *C4B*, *C5*, *CFD*, *CFI*, *FPR1*…

### Wnt signaling pathway analysis

**Figure 1.**
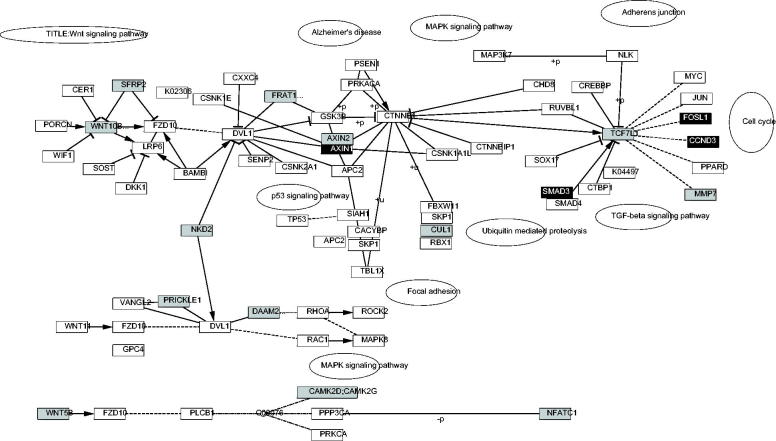
The Wnt signaling pathway. Black blocks represent up-regulated genes, and gray blocks represent down-regulated genes.

According to Figure 1, four up-regulated genes [*FOSL1* (FOS-like antigen 1), *SMAD3* (SMAD family member 3), *AXIN1* (axin 1), and *CCND3* (cyclin D3)] and 14 down-regulated genes [e.g., *NFATC3* (nuclear factor of activated T cells (NFAT), cytoplasmic 3), and *TCF7L1* (TF 7-like 1)] participated in the Wnt signaling pathway.

### Transcriptional regulatory network analysis

**Figure 2.**
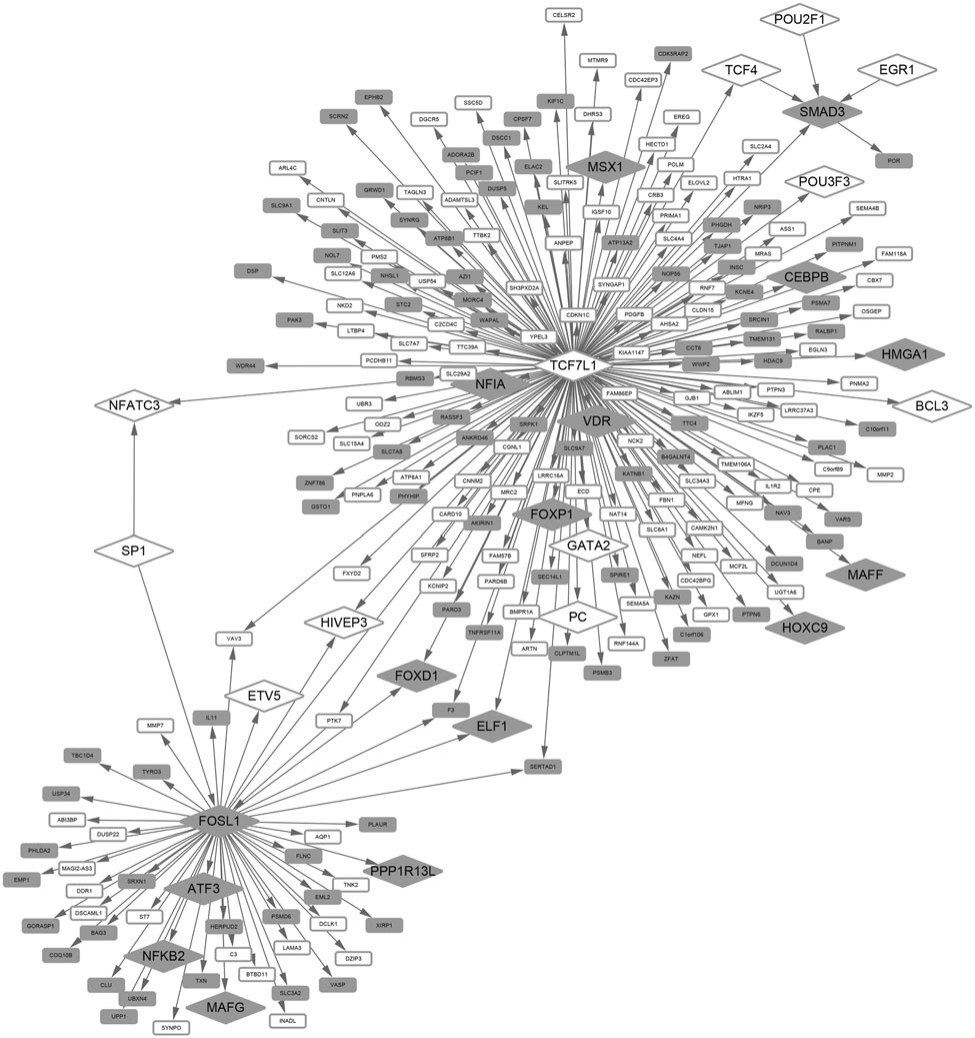
The transcriptional regulatory network composed of *SMAD3*, *FOSL1*, *NFATC3*, and *TCF7L1*, as well as their target genes. Gray nodes represent up-regulated genes; white nodes represent down-regulated genes; diamonds represent transcription factors, and rounded rectangles represent target genes.

A total of 69 TFs were identified from the DEGs, including 32 up- and 37 down-regulated TFs. Furthermore, two up-regulated TFs (*SMAD3* and *FOSL1*) and two down-regulated TFs (*NFATC3* and *TCF7L1*) were involved in the Wnt signaling pathway. Additionally, there were 238 nodes (112 up- and 126 down-regulated DEGs) and 247 edges in the transcriptional regulatory network. Among the 238 DEGs, there were 27 TFs, and *TCF7L1* was the hub, regulating a set of genes and other TFs (e.g., *NFATC3* and *SMAD3*) (Figure 2).

### Pathway-crosstalk analysis

**Figure 3.**
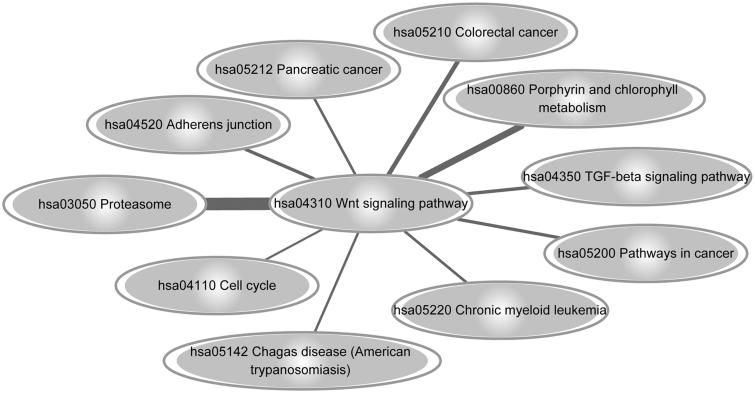
The pathway crosstalk between Wnt signaling pathway and other pathways (top 10 at weight scores). The thickness of the edges is directly proportional to the significance of interaction between any two pathways.

**Figure 4.**
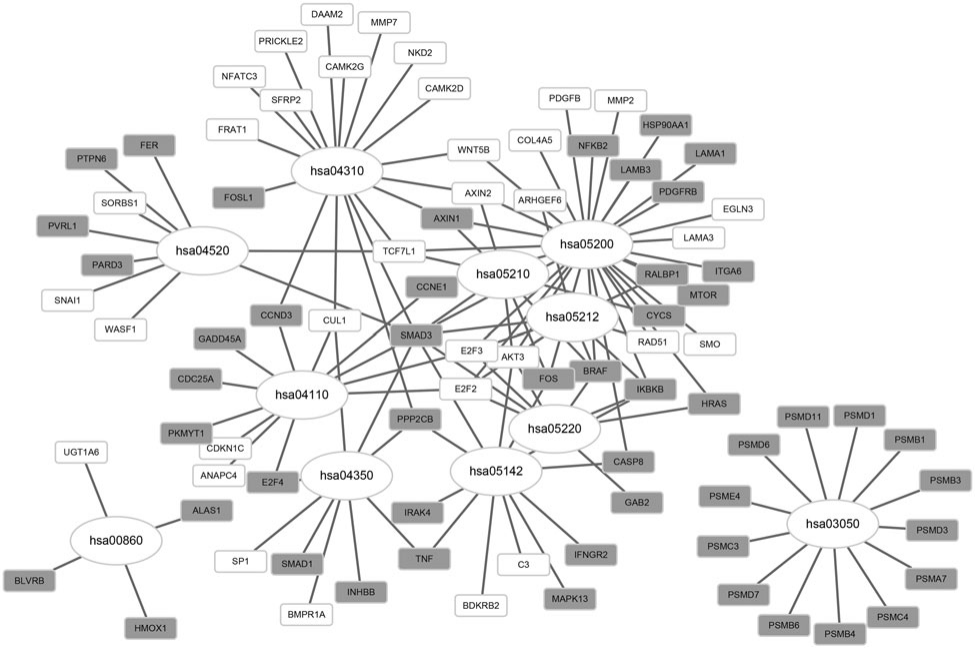
Network of crosstalk pathways and their related differentially expressed genes. Gray rectangles represent up-regulated genes; white rectangles represent down-regulated genes; and ellipses represent pathways.

In total, 211 pathways were predicted to be linked to Wnt signaling pathway. Among the pathways, the pathway of proteasome (hsa03050) was most closely linked to the Wnt signaling pathway (hsa04310) (Figure 3). A series of up-regulated genes were enriched in the proteasome pathway, such as *PSMA7*, *PSMB1* and *PSMB3*. The up-regulated genes *SMAD3* was enriched in multiple pathways, such as cell cycle (hsa04110), pathways in cancer (hsa05200), and adherens junction (hsa04520) (Table 4). Based on the DEG annotation, a set of DEGs were involved in the crosstalk of Wnt and other signaling pathways, such as *TCF7L1*, *SMAD3* and *FOSL1* (Figure 4).

**Table 4.** The differentially expressed genes enriched in the crosstalk pathways of the Wnt signaling pathway.

Pathway	Differentially expressed genes
hsa04110	CCND3*, CCNE1*, CDC25A*, E2F4*, GADD45A*, MERTK*, RALBP1*, RRP9*, SART1*, SMAD3*
hsa05200	BRAF*, BRPF1*, CASP8*, CCNE1*, FOS*, HRAS*, HSP90AA1*, IKBKB*, ITGA6*, LAMB3*, MTOR*, NFKB2*, PDGFRB*, RRP9*, SERGEF*, SMAD3*, WRN*
hsa05220	BRAF*, HRAS*, IKBKB*, SMAD3*
hsa00860	ALAS1*, BLVRB*, HMOX1*, *HHIPL2*, *INO80*, *UGT1A6*
hsa03050	PSMA7*, PSMB1*, PSMB3*, PSMB4*, PSMB6*, PSMC3*, PSMC4*, PSMD1*, PSMD11*, PSMD3*, PSMD7*
hsa04310	*FRAT1*, *CAMK2G*, *MMP7*, *WNT5B*, *WNT10B*, *NFATC3*, *AXIN2*, *TCF7L1*, *PRICKLE2*, *CAMK2D*, *SFRP2*, *NKD2*, *DAAM2*, *CUL1*
hsa04350	E2F4*, INHBB*, PPP2CB*, SMAD1*, SMAD3*
hsa05142	CASP8*, FOS*, IFNGR2*, IKBKB*, MAPK13*, PHLPP1*, PPP2CB*, SMAD3*
hsa04520	FER*, PTPN6*, PVRL1*, RPS6KA4*, SMAD3*
hsa05210	BRAF*, FOS*, SMAD3*
Hsa05212	BRAF*, IKBKB*, SMAD3*, *E2F2*, *E2F3*, *RAD51*

*Up-regulated genes.

## Discussion

As_2_O_3_, as a treatment for cancers, has been studied for several decades. However, the molecular mechanisms of As_2_O_3_ treatment in renal cancer remains not clear, especially the effect of As_2_O_3_ on the Wnt signaling pathway. In this study, 1684 DEGs between As_2_O_3_ treatment samples and controls were identified, including 821 up- and 863 down-regulated genes. Among those DEGs, *SMAD3*, *FOSL1*, *NFATC3* and *TCF7L1* were identified as TFs and predicted to be associated with the Wnt signaling pathway.

*SMAD3* encodes a member of the smad family. SMAD proteins are signal transducers and transcriptional modulators that are activated by TGF-β and mediate multiple signaling pathways [23]. Crosstalk between Smad and Wnt signaling has been reported in several kinds of cancer, such as pancreatic ductal adenocarcinoma and hepatocellular carcinoma [24], as well as kidney fibrosis [25]. In this study, according to the results of enrichment analyses, *SMAD3* was enriched in the functions of cell death and apoptotic process and a set of Disease Ontology terms about cancer. TGF-β acts as a tumor suppressor during the early stages of tumorigenesis, and the ability of TGF-β/SMAD3 signal in tumor suppression has been demonstrated by multiple studies [26–28]. A recent study has demonstrated that nuclear expression of Smad3 is closely related to prognosis of clear cell RCC patients [29]. Taken together, As_2_O_3_ may play an anti-apoptosis role in renal cancer by up-regulating the expression of *SMAD3* as well as the pathway crosstalk between TGF-β/SMAD3 and Wnt signaling pathway.

*FOSL1* encodes fos-like antigen 1, also known as *Fra1*, a member of Fos gene family, which regulates cell proliferation, differentiation, and transformation [30]. *FOSL1* can function as either an activator or a repressor to control the equilibrium between migration and adhesion in sprouting angiogenesis, which is critical in tumorigenesis [31,32]. In this study, *FOSL1* was up-regulated in the 786-O cells with As_2_O_3_ treatment, while a study has reported that Fosl1 induces transformation and invasiveness of human epithelial adenocarcinoma cells [33], which is inconsistent with the result of this study. It may be due to that during the early stage of As_2_O_3_ treatment, the expression of *FOSL1* is not effectively influenced, and with the prolonged time of As_2_O_3_ treatment, the expression of *FOSL1* may be decreased, which is needed to be further investigated.

*NFATC3* encodes a member of the nuclear factors of activated T cells DNA-binding transcription complex, and it is important for T-cell development and essential for cancer chemoresistance [34]. NFAT proteins are able to inhibit the Wnt/β-catenin pathway via participating in regulating cell proliferation and differentiation [35,36]. Study has reported that NFAT signaling controls nephron formation, and *NFATC3* is abundantly expressed in the metanephric mesenchyme [37]. In *de novo* renal allograft recipients, expression levels of NFAT-regulated genes are closely related to the clinical outcomes [38]. Furthermore, NFAT has been recently demonstrated to play pivotal roles in kidney ischemia/reperfusion (I/R) injury [39]. There is no evidence to support the association of *NFATC3* with renal cancer so far, while the increased *NFATC3* expression has been detected in human angiosarcoma that was induced by secreted frizzle-related protein 2 (SFRP2) [40]. Collectively, As_2_O_3_ treatment may affect the Wnt/β-catenin pathway through decreasing the expression of *NFATC3* in renal cancer.

*TCF7L1* (also known as *TCF3*) encodes a member of the T-cell factor/lymphoid enhancer factor family of TFs, which are activated by β-catenin and mediate the Wnt signaling pathway [41]. A previous study has demonstrated that the expression of *TCF1* that is the homolog of *TCF7L1* is significantly higher in clear cell RCC than in normal tissue [42]. Besides, in the transcriptional regulatory network, *TCF7L1* regulated other TFs, such as *SMAD3* and *NFATC3*. Taken together, the Wnt signaling pathway may be affected by As_2_O_3_ in renal cancer via the reduced expression of *TCF7L1*.

Furthermore, in this study, the pathway of proteasome (hsa03050) was most closely related to the Wnt signaling pathway, and the proteasome pathway was significantly enriched by a set of up-regulated DEGs that encode subunits of proteasome, such as *PSMA7* and *PSMB1*. Proteasomes, along with ubiquitin (Ub), are essential components of the energy-dependent, nonlysosomal proteolytic pathway. β-catenin in the Wnt signaling pathway exerts functions in cells depending on the proteasome [43]. The levels of mRNAs for the subunits of proteasomes are high in rapidly proliferating renal cancer cells [44]. Besides, study has reported that *PSMB1* is highly expressed in RCCs, comparing with the normal kidney tissues [45]. Therefore, the Wnt signaling pathway may be influenced by As_2_O_3_ in renal cancer through the proteasome pathway.

Despite the aforementioned results, there were still some limitations in this study. The predicted results, such as expression of the discussed genes, and the associations between genes and Wnt signaling pathway, were required to be confirmed by experiments in renal cancer tissues, which would be conducted in our future study and reported separately.

## Conclusions

In conclusion, 1684 DEGs (821 up- and 863 down-regulated genes) between As_2_O_3_ treatment samples and controls were identified. Among them, *SMAD3*, *FOSL1*, *NFATC3* and *TCF7L1* were identified as TFs and predicted to be correlated with the Wnt signaling pathway. Furthermore, the pathway of proteasome was most closely linked to the Wnt signaling pathway. These genes and pathways may play pivotal roles in the As_2_O_3_ treatment of renal cancer. These findings may provide new information for the molecular mechanisms of As_2_O_3_ treatment on renal cancer, especially the influence of As_2_O_3_ on the Wnt signaling pathway in renal cancer.
